# Intramuscular Exposure to a Lethal Dose of Ricin Toxin Leads to Endothelial Glycocalyx Shedding and Microvascular Flow Abnormality in Mice and Swine

**DOI:** 10.3390/ijms222212345

**Published:** 2021-11-16

**Authors:** Anita Sapoznikov, Yoav Gal, Yentl Evgy, Moshe Aftalion, Shahaf Katalan, Tamar Sabo, Chanoch Kronman, Reut Falach

**Affiliations:** 1 Department of Biochemistry and Molecular Genetics, Israel Institute for Biological Research, Ness Ziona 74100, Israel; yoavg@iibr.gov.il (Y.G.); yentle@iibr.gov.il (Y.E.); moshea@iibr.gov.il (M.A.); tamarsa@gmail.com (T.S.); kronmanc@gmail.com (C.K.); reutf@iibr.gov.il (R.F.); 2 Division of Medical Chemistry, Israel Institute for Biological Research, Ness Ziona 74100, Israel; shahafk@iibr.gov.il

**Keywords:** ricin, intramuscular, glycocalyx shedding, heparan sulfate, hyaluronic acid, syndecan-1, microcirculation

## Abstract

Ricin toxin isolated from the castor bean (*Ricinus communis*) is one of the most potent and lethal molecules known. While the pathophysiology and clinical consequences of ricin poisoning by the parenteral route, i.e., intramuscular penetration, have been described recently in various animal models, the preceding mechanism underlying the clinical manifestations of systemic ricin poisoning has not been completely defined. Here, we show that following intramuscular administration, ricin bound preferentially to the vasculature in both mice and swine, leading to coagulopathy and widespread hemorrhages. Increased levels of circulating VEGF and decreased expression of vascular VE-cadherin caused blood vessel impairment, thereby promoting hyperpermeability in various organs. Elevated levels of soluble heparan sulfate, hyaluronic acid and syndecan-1 were measured in blood samples following ricin intoxication, indicating that the vascular glycocalyx of both mice and swine underwent extensive damage. Finally, by using side-stream dark field intravital microscopy imaging, we determined that ricin poisoning leads to microvasculature malfunctioning, as manifested by aberrant blood flow and a significant decrease in the number of diffused microvessels. These findings, which suggest that glycocalyx shedding and microcirculation dysfunction play a major role in the pathology of systemic ricin poisoning, may serve for the formulation of specifically tailored therapies for treating parenteral ricin intoxication.

## 1. Introduction

Ricin, a highly toxic protein derived from the bean of the castor plant (*Ricinus communis*), consists of two polypeptide chains linked by a disulfide bond. While the B-chain lectin is responsible for cell binding and entry, the A-chain has the ability to depurinate the 28S RNA component of eukaryotic ribosomes in a site-specific manner and thereby block cell protein synthesis [1]. Ricin is considered a high-priority biothreat agent by the Centers for Disease Control and Prevention (CDC) due to its accessibility, ease of production, stability and extreme toxicity [2]. Exposure to ricin can occur by oral ingestion, inhalation or parenteral intoxication, while the features and severity of ricin toxicity vary markedly with the route of exposure. Thus, inhalatory and parenteral intoxications are associated with greater illness and mortality, and are characterized by lethal doses that are roughly three magnitudes lower than that of oral poisoning [3]. In this study, we focused on parenteral intoxication, as, in recent years, several terrorist attempts to utilize weaponized ricin delivered by sharp or explosive devices were planned [4,5]. When delivered by the parenteral route, ricin is systemically distributed throughout the body, and post-mortem examinations in a single well-known case involving the assassination of Georgi Markov, suggested that cardiac arrest due to necrosis of the cardiac conducting tissue may be the direct cause of death [6]. In the murine model of parenteral ricin intoxication, we have previously documented that, in addition to thrombocytopenia, coagulopathy and multi-organ hemorrhages, profound ricin-induced morpho-pathological and functional damage in the spleen, bone marrow and cardiovascular system also occurs [7]. In the swine animal model, intramuscular exposure to ricin entailed the development of a severe distributive shock, characterized by severe vasodilation, low central vein oxygen saturation and an increase in the venous-to-arterial carbon dioxide difference, indicating an increase in tissue oxygen demand not met by cardiac supply [8].

The endothelial glycocalyx lines the luminal surface of the vascular endothelium and is composed of membrane-bound glycoproteins and negatively charged proteoglycans (e.g., syndecans) associated with various glycosaminoglycans (GAGs), such as heparan sulfate (HS) and hyaluronic acid (HA) [9]. The glycocalyx regulates vascular permeability and vascular tone, inhibits microvascular thrombosis and helps regulate leukocyte adhesion on the endothelium [10,11,12]. During sepsis, which is a form of distributive shock, the glycocalyx is degraded via inflammatory mechanisms, such as metalloproteinase- and sheddase-mediated proteolysis, which, in turn, leads to vascular hyperpermeability, unregulated vasodilation, microvessel thrombosis and augmented leukocyte adhesion. Glycocalyx fragments shed into the blood during sepsis are considered clinically relevant biomarkers of endothelial dysfunction and are correlated with disease severity [13,14,15]. Moreover, degradation of the glycocalyx is thought to contribute to microcirculatory dysfunction in sepsis [16]. Specifically, sepsis-induced alterations in sublingual microcirculatory functioning were found to be highly correlated with poor prognosis [17].

In the present study, we investigated the mechanisms of vascular injury following exposure of mice and swine to a lethal dose of ricin by the intramuscular route. Our findings, which demonstrated the involvement of glycocalyx shedding and microcirculation dysfunction in the aberrant coagulation and vascular hyperpermeability that occurred in the wake of systemic ricin poisoning, may serve as a basis for the formulation of specific adjunct therapies for parenteral ricin intoxication.

## 2. Results

### 2.1. Ricin Binds to Blood Vessels and Promotes Vascular Permeability of Mouse Tissues

**Figure 1:**
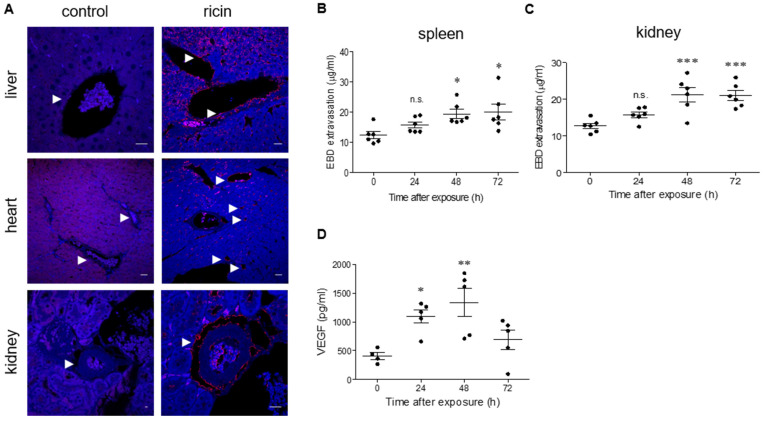
Ricin binding and organ hyperpermeability in ricin-intoxicated mice. (**A**) Immunofluorescence (red) of liver, heart and kidney sections before (control) or 30 min after incubation with ricin (5 µg/mL). The arrows indicate blood vessels and ricin binding. Nuclei stained by DAPI appear in blue. Scale bar: 20 µm (representative sections of *n* = 3 mice/group are shown). EBD in the spleen (**B**) and kidneys (**C**) following ricin intoxication. Control (0 h) or ricin-intoxicated mice (2LD_50_ i.m., 18 µg ricin/kg body weight) were intravenously injected with 50 mg/kg EBD at the indicated time points and tissues were harvested 1 h later and monitored for EBD content. Each dot represents an individual mouse. (**D**) Circulating VEGF levels were determined in the serum samples from non-intoxicated (0 h) and ricin-intoxicated mice at the indicated time points. Each dot represents an individual mouse. In (**B**–**D**), the results are depicted as means ± SEM. Statistical analyses were performed by one-way ANOVA followed by Dunnett’s post hoc test. * *p* < 0.05; ** *p* < 0.01; *** *p* < 0.001; n.s., not significant.

Previous studies demonstrated that intramuscular ricin poisoning of mice induces prompt coagulopathy with continuous prolongation of prothrombin (PT) and activated partial thromboplastin (APTT) times and an elevation of circulatory fibrinogen in parallel with severe thrombocytopenia. Multi-organ hemorrhages and morphological and functional damages were detected in the spleen, bone marrow and heart, yet these changes did not correlate with the respective cytotoxic ricin-dependent catalytic activity measured in the damaged organs. To further understand the nature of parenteral ricin intoxication, we monitored ricin binding in various tissues. To this end, sections from various mouse tissues were subjected to short-term incubation with ricin, and the tissue-bound toxin was then visualized by incubation with anti-ricin antibody, followed by staining of the sections with fluorescent secondary antibody. In the liver, ricin staining was detected in the veins and sinusoidal walls. In the heart as well, ricin was found to bind to large and small vessels. Ricin staining in the kidney was also confined to blood vessels (Figure 1A). Previously, myocardium hyperpermeability was documented in mice following intramuscular exposure to ricin. Since we observed ricin binding in the blood vessels of various tissues, we tested whether other organs, in addition to the heart, became hyperpermeabilized. Using the Evans Blue dye (EBD) extravasation assay, we observed a consistent elevation in hyperpermeability in the spleen (Figure 1B) and kidneys (Figure 1C), with the most pronounced effect at 48–72 h after exposure. In line with this observation, the levels of vascular endothelial growth factor (VEGF), a factor well-known to increase endothelial permeability [18], were found to be upregulated in the serum of ricin-intoxicated mice at 24 h after exposure and even more so at 48 h post-exposure (Figure 1D).

### 2.2. Intramuscular Exposure of Mice to Ricin Leads to Impairment of the Vascular Glycocalyx

**Figure 2:**
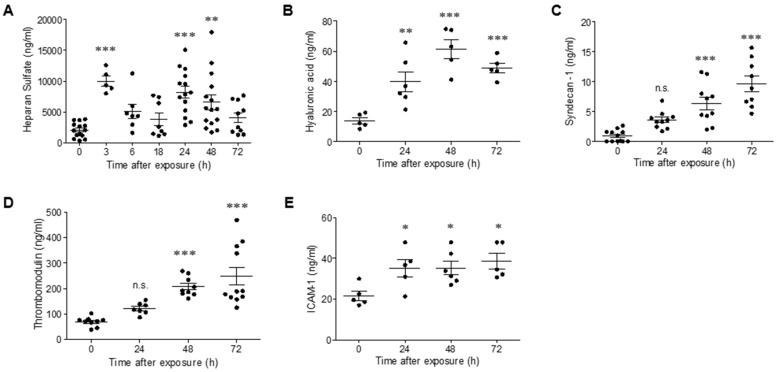
Degradation of the glycocalyx in ricin-intoxicated mice. Circulating levels of HS (**A**), HA (**B**), syndecan-1 (**C**), thrombomodulin (**D**) and ICAM-1 (**E**) were determined in serum samples collected from non-intoxicated (0 h) and ricin-intoxicated (2LD_50_ i.m., 18 µg ricin/kg) mice at the indicated time points. Each dot represents an individual mouse. The results are depicted as means ± SEM. Statistical analyses were performed by one-way ANOVA followed by Dunnett’s post hoc test. * *p* < 0.05; ** *p* < 0.01; *** *p* < 0.001; n.s., not significant.

High-level expression of pro-inflammatory mediators (e.g., interleukin 1 β (IL-1β), IL-6 and tumor necrosis factor α (TNFα)) and VEGF has been shown to promote leukocyte migration and to impair the vascular glycocalyx, which plays a vital role in the regulation of endothelial permeability [19]. Since the elevation of pro-inflammatory cytokines and increased leukocytes in blood counts were also previously documented following intramuscular ricin poisoning of mice [7], we examined whether degradation of the glycocalyx may play a role in ricin-induced vascular permeability. Degradation of the glycocalyx results in the shedding of soluble compounds that can be quantified in the serum following their release from the endothelium to the bloodstream. Following intramuscular exposure of mice to ricin, in serum samples, we observed a two-peak wave of soluble heparan sulfate (HS), the predominant glycosaminoglycan (GAG) in the glycocalyx layer; the first peak appeared as early as 3 h post-exposure, followed by a second peak at 24 h after ricin intoxication (Figure 2A). Monitoring of an additional GAG, hyaluronic acid (HA), demonstrated elevated serum levels at 24 h after exposure, which continued to increase at later time points 48–72 h post-exposure (Figure 2B). We further measured the soluble form of syndecan-1, a transmembrane core protein of the glycocalyx whose shedding is associated with coagulopathy [20]. Release of this component was evident at 48 h, with further elevation at 72 h post-exposure (Figure 2C), thus correlating with the previously reported elevation in PT and APTT in this model of ricin poisoning. Damage of the endothelial cells induced the release of thrombomodulin in its soluble form, which is normally interwoven in the glycocalyx network [21]; indeed, we observed elevated thrombomodulin levels in serum samples collected 48–72 h after ricin exposure (Figure 2D).

Previous reports suggesting that glycocalyx shedding leads to the release of adhesion molecules to the circulation [22] prompted us to measure the levels of the adhesion molecule ICAM-1 in serum samples collected at various time points from ricin-intoxicated mice. Indeed, high levels of soluble ICAM-1 were measured 24 h after ricin exposure and also persisted at later time points (Figure 2E). Altogether, these data are indicative of profound ricin-induced damage to the structure of the endothelium and its glycocalyx coating, which, in turn, may compromise endothelial permeability and function.

### 2.3. Systemic Ricin Intoxication of Swine Leads to Coagulopathy and Multi-Organ Hemorrhages

**Figure 3:**
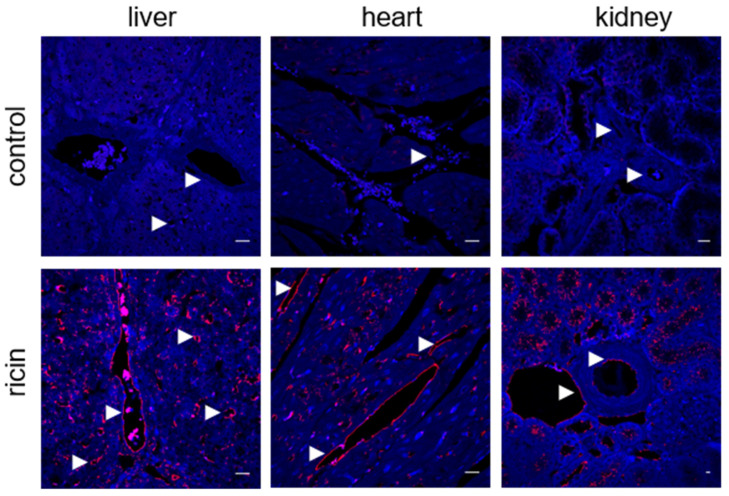
Ricin binding to swine tissues. Immunofluorescence (red) of the liver, heart and kidney sections before (control) or 30 min after incubation with ricin (5 µg/mL). The arrows indicate blood vessels and ricin binding. Nuclei stained by DAPI appear in blue. Scale bar: 20 µm (representative sections of *n* = 3 swine/group are shown).

Since large animal models such as swine have greater bench-to-bedside translational potential due to their prominent similarity to humans in terms of anatomy, genetics and physiology [23], we further evaluated the effects of intramuscular ricin poisoning on the vasculature of swine. As in the mouse model, short-term incubation of swine tissues with the toxin demonstrated ricin binding, mainly to different blood vessels in the liver, heart and kidneys (Figure 3).

**Figure 4:**
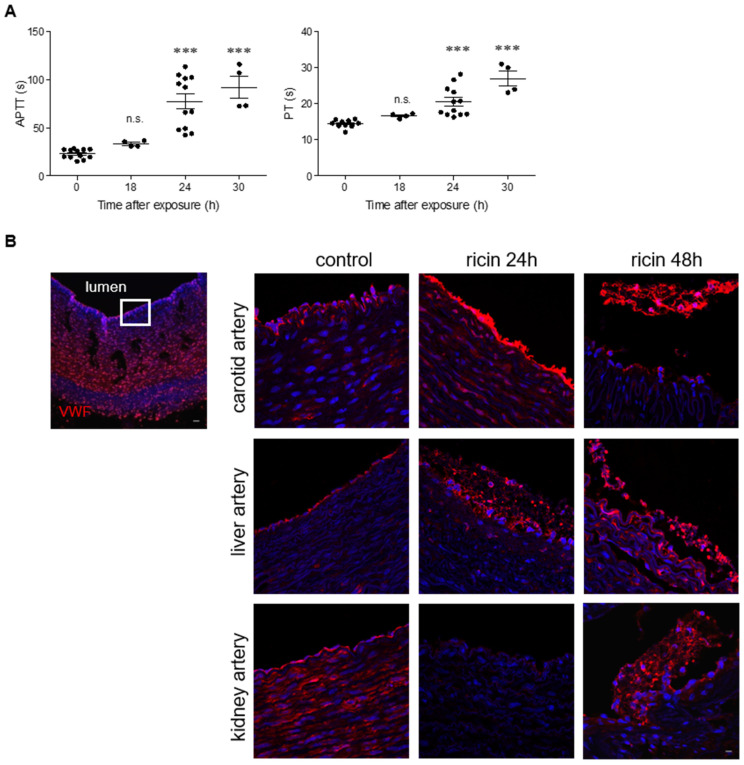

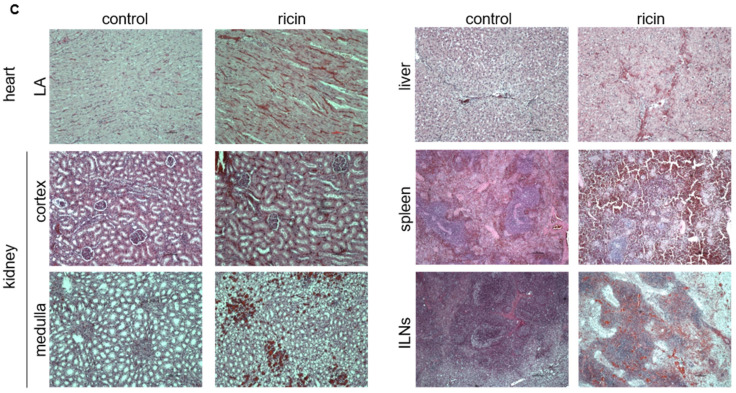
Coagulation parameters and histological analysis of tissues from ricin-intoxicated swine. (**A**) APTT and PT were determined in peripheral blood samples collected from control pigs (0 h) or at the indicated time points after i.m. exposure to a lethal dose of ricin (7.5 µg ricin/kg body weight); s = seconds. Each dot represents an individual pig. The results are depicted as means ± SEM. Statistical analyses were performed by one-way ANOVA followed by Dunnett’s post hoc test. *** *p* < 0.001; n.s., not significant. (**B**) Immunofluorescence (red) shows VWF staining on the apical side of swine arteries in control animals or 24 and 48 h after ricin exposure. Images on the right are enlargements of the apical side of the artery (as indicated by the square on the left image); the lumen of the vessel is always upwards. Nuclei stained by DAPI appear in blue. Scale bar: 20 µm (representative sections of *n* = 3 swine/group are shown). (**C**) Sections of the heart (left atrium, LA), cortex and medulla regions of the kidney, liver, spleen and ILNs from non-intoxicated (control) and ricin-intoxicated pigs at 24 h post-exposure. The sections were stained with hematoxylin and eosin (H&E). Scale bar: 100 µm (representative sections of *n* = 3 swine/group are shown).

Coagulopathy was previously determined only in the murine model of intramuscular ricin poisoning; therefore, we now examined the coagulation rates in swine following injection of a lethal dose of ricin into the thigh muscle. Analysis of plasma samples collected at various time points revealed a prolongation of APTT and PT at 24 h post-exposure and onward (Figure 4A). In addition to the observed coagulopathy, ricin-intoxicated swine displayed thrombocytopenia [8], a pathological state that was also previously shown in parenterally intoxicated mice. We therefore checked for alterations in the expression of von Willebrand factor (VWF), a large adhesive glycoprotein required for platelet adhesion to the subendothelium at the site of vessel injury, which is involved in platelet aggregation and the formation of platelet plugs [24]. At 24 h after intramuscular exposure to ricin, exposed VWF was observed on the lumen of the carotid, while, at the same time point, long fibers of VWF bound to platelets were visualized in the liver artery. At 48 h post-exposure, networks of elongated fibers and platelets were abundant in the lumen of different blood vessels in the liver and in the kidneys (Figure 4B), presumably as part of the process of coagulation and inflammation. This process could also explain the observed platelet consumption and thrombocytopenia. In addition to aberrant coagulation and in agreement with the ostensible vascular damage, hemorrhagic areas were observed in various organs 24 h after intoxication of swine with ricin. In the heart, the bleeding was mostly confined to the left atrium (LA) (Figure 4C), whereas in the right atrium (RA), only minute bleeding was detected and mild myocyte damage was observed. No pathological alterations could be detected in the left ventricle (LV) (Figure S1). In the kidneys, hemorrhages were discerned in the cortex and in the medulla, and additional bleeding areas were noticed in the liver. In contrast to the small-scale lesions exhibited in the abovementioned tissues, widespread damage was observed in the spleen and inguinal lymph nodes (ILNs). Atrophy of the white pulp in the spleen, and decreased cellularity, necrosis and hemorrhage were observed in both tissues (Figure 4C). This body of data indicates that following intramuscular exposure to ricin, swine develop coagulopathy and multi-organ hemorrhage as a result of vascular damage.

### 2.4. Ricin Intoxication Promotes Shedding of Glycocalyx Components in Swine

**Figure 5:**
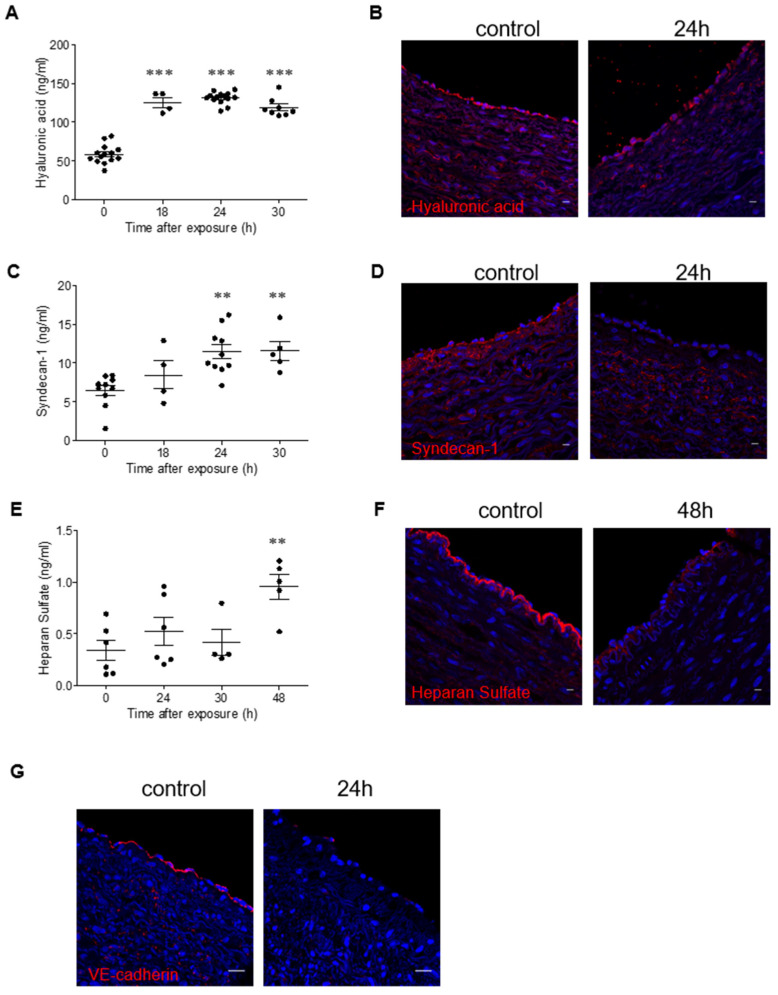
Shedding of the glycocalyx in ricin-intoxicated swine. (**A**,**C**,**E**) Circulating levels of HA (**A**), syndecan-1 (**C**) and HS (**E**) determined in control animals and in ricin-intoxicated animals at the indicated time points after intramuscular exposure (7.5 µg/kg). Each dot represents an individual pig. (**B**,**D**,**F**,**G**) Immunofluorescence (red) of HA (**B**), syndecan-1 (**D**), HS (**F**) and VE-cadherin (**G**) in the apical side of swine arteries of control animals and of ricin-intoxicated animals at the indicated time points after intramuscular exposure (7.5 µg/kg). The lumen of the vessel is always upwards. Nuclei stained by DAPI appear in blue. Scale bar: 20 µm (representative sections of *n* = 3 swine/group are shown). The results are depicted as means ± SEM. Statistical analyses were performed by one-way ANOVA followed by Dunnett’s post hoc test. ** *p* < 0.01; *** *p* < 0.001.

As endothelial glycocalyx disruption may contribute to coagulopathy, to have a measure of the intensity of damage to the vascular system, we monitored blood samples collected from ricin-intoxicated swine for the presence of soluble glycocalyx components. Elevated HA levels were determined at 18 h after exposure (Figure 5A). Immunostaining of HA on the endothelium of swine blood vessels revealed that the intact impression observed in naïve pigs (0 h) was disrupted at 24 h following intoxication (Figure 5B). Analysis of syndecan-1 showed that the levels of the soluble form of this proteoglycan were elevated at 24 h after exposure to ricin and remained high at 30 h post-exposure (Figure 5C). These results were further corroborated by immunostaining experiments, which demonstrated the disappearance of bound syndecan-1 from the vascular endothelial sheet of intoxicated swine at 24 h post-exposure (Figure 5D). Measurement of soluble HS in blood samples revealed more delayed damage that was evidenced only at 48 h after intoxication (Figure 5E); the same damage could be also demonstrated by immunostaining of swine blood vessels at the same time point (Figure 5F). As vascular permeability is governed not only by the intact glycocalyx but also by intercellular junction proteins, we monitored the expression of VE-cadherin on the endothelial cells of ricin-intoxicated swine and found reduced expression of this protein at 24 h post-exposure (Figure 5G). These results indicate that, as in mice, following intramuscular ricin exposure of swine, there is a substantial damage to the blood vessels, which is manifested as glycocalyx shedding, not only of the GAGs that bind to the exterior surface of the proteoglycans (i.e., HS) or to membranal receptors (such as HA), but also of core proteoglycans (such as syndecan-1), which are anchored to the apical membrane of endothelial cells. This vascular damage is not limited to the glycocalyx, but also includes damage to the intercellular junctions, both of which contribute to vessel injury, hyperpermeability and aberrant communication between endothelial cells.

### 2.5. Intramuscular Ricin Intoxication Promotes the Impairment of Microcirculation in Mice and Swine

**Figure 6:**
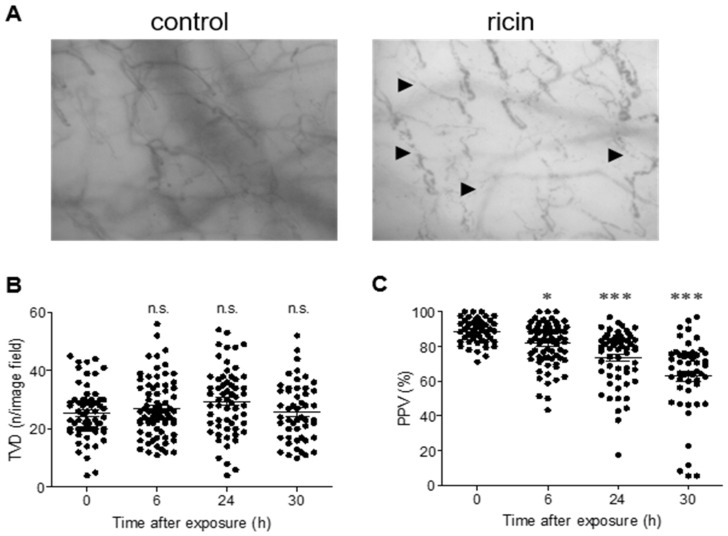

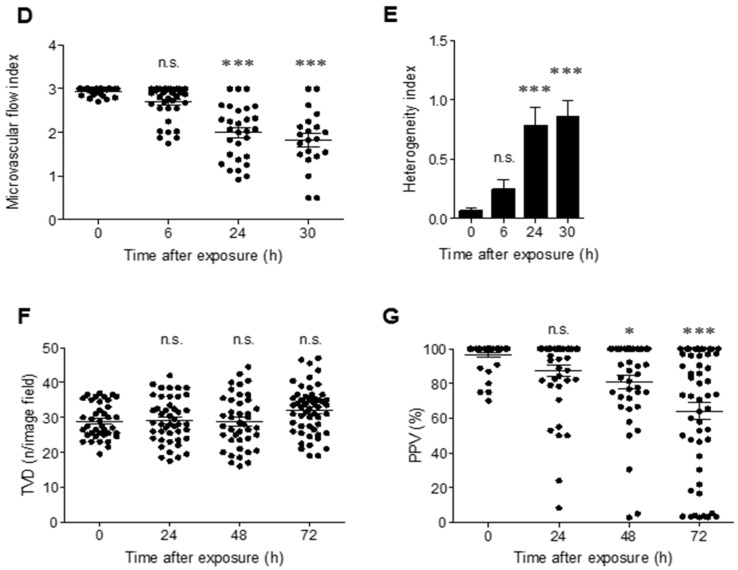
Measurement of microcirculation functionality in ricin-intoxicated swine and mice. (**A**) Representative SDF images of the sublingual microcirculation in swine before (control) or 30 h after ricin exposure (7.5 µg/kg). The arrows indicate areas with impaired microvascular flow. Microcirculatory parameters TVD (**B**), PPV (**C**), MFI (**D**) and HI (**E**) were determined in swine before (0 h) or at the indicated time points after ricin exposure. (*n* = 5 in each group; each dot represents data from an individual movie). TVD (**F**) and PPV (**G**) were determined in the cecum of mice before (0 h) or at the indicated time points after ricin exposure (18 µg/kg) (*n* = 5 in each group; each dot represents data from an individual movie). The results are depicted as means ± SEM. Statistical analyses were performed by one-way ANOVA followed by Dunnett’s post hoc test. * *p* < 0.05; *** *p* < 0.001; n.s., not significant.

The endothelial glycocalyx layer serves as an active interface between the blood and the capillary wall, and the intravascular lumen of blood vessels comprises both the non-circulating glycocalyx volume and the circulating plasma. Shedding of the glycocalyx and compaction of this layer increases plasma volume, thereby leading to the dilution of red blood cells [12]. Having established that glycocalyx shedding occurs following intramuscular application of ricin, we continued to examine microcirculatory functionality in ricin-intoxicated animals by using the side-stream dark field (SDF) imaging technique [25]. To this end, swine were subjected to sublingual microcirculation analysis before and at 6, 24 and 30 h after ricin exposure. Visual changes could be observed: the microvessels seemed to be both dilated and discontinuous, resulting in a dotted-line appearance characteristic of impaired microvascular flow (Figure 6A, Videos S1–S3). Though quantitative measurements of the sublingual microcirculation did not show alterations in total vessel density (TVD) (Figure 6B), a significant decrease in the proportion of perfused vessels (PPV) was observed as early as 6 h after exposure of swine to ricin and was even more pronounced at 24 h and 30 h post-exposure (Figure 6C). Moreover, at 24 h after intoxication, the microvascular flow index (MFI), a measure of the velocity of blood flow, had already dropped from a score of 3 (normal continuous flow before intoxication) to 2 (sluggish flow), with some vessels exhibiting no flow at all (a score of 1) at 24 and 30 h after intoxication (Figure 6D). We thus calculated the heterogeneity index (HI), a parameter reflecting differences in the flow velocity in different sublingual areas of the same animal at a given time point. A trend of elevated HI, albeit not statistically significant, was already in evidence at 6 h after exposure. At 24 and 30 h after intoxication, HI was statistically high (Figure 6E), indicating impaired vascular perfusion and substantially flawed microcirculatory performance.

Measurements of the microcirculation of mice following ricin intoxication were performed by imaging the cecum of naïve animals and mice at 24, 48 and 72 h after ricin intoxication. Since the SDF imaging apparatus was adjusted to a greater degree for larger subjects (humans and swine), in mice, only the parameters of TVD and PPV could obtained. In a similar manner to the results in swine, no alteration in the TVD of small vessels was detected (Figure 6F), while decreased PPV was observed. Specifically, at 48 h after ricin exposure, there was a statistically significant drop in PPV that was even more pronounced at 72 h post-exposure (Figure 6G). Altogether, the results in both the murine and swine models suggested that the animals suffered from ongoing hypoperfusion and increased flow heterogeneity, which progressively worsened with time.

## 3. Discussion

The multi-faceted pathophysiological disorders related to systemic ricin exposure have been the focus of some recent studies. In the murine model, multi-organ hemorrhages and thrombocytopenia, as well as severe spleen, bone marrow and cardiovascular damage, were observed [7]. In a large animal model, swine were shown to develop distributive shock, characterized by pronounced vasodilatation (decreased systemic vascular resistance), low central venous oxygen saturation and escalating ΔPCO_2_ (venous CO_2_-to-arterial CO_2_) values, indicating an increase in tissue oxygen demand that was not met by cardiac supply [8]. In a few recent cases of suicide attempts by parenteral ricin injection in humans, the patients suffered from thrombocytopenia and coagulopathy, hypovolemic shock and cardiorespiratory collapse, and they eventually died due to multiple organ failure [26,27]. However, the molecular mechanisms leading to these clinical manifestations were not explored and basic facts regarding the biodistribution profiles of intramuscularly injected toxin and the identity of the toxin targets within the organism have not yet been fully appreciated. To address these issues, we first monitored the binding of ricin to tissue sections prepared from mice and swine following i.m. intoxication. In agreement with other studies [28,29,30], we detected prompt and preferential binding of ricin to the vasculature in all tissues examined, which correlated with markedly increased tissue permeability in organs, such as the spleen and kidneys and, as previously published, the heart. Interestingly, despite the preferential binding of ricin to the vasculature in the liver, we could not detect hyperpermeability in this organ (data not shown) and only minor histological changes were observed. However, it could be that these changes were sufficient for significant serum elevation of alanine transaminase (ALT) and alkaline phosphatase (ALP) following intramuscular exposure of mice to ricin, which are markers of liver damage [7].

High levels of VEGF, a factor known to provoke vascular permeability by uncoupling endothelial cell–cell junctions, were measured in the serum of the intoxicated animals, and loss of the VE-cadherin junction protein was readily documented in swine following ricin application. Upon the occurrence of alleged VEGF-mediated vascular leak, the exposed underlying basement membrane can attract platelets which bind to von Willebrand factor, fibronectin or underlying collagen, leading to their activation. These activated platelets often deposit high levels of VEGF, thus activating a vicious circle whereby the leakage is extended, leading to additional cycles of platelet recruitment and VEGF overexpression [31]. In agreement with this notion, in ricin-intoxicated swine, we observed the secretion of VWF from the endothelium in the form of fiber networks, which were found to be associated with platelets. Release of the VWF that is stored in Weibel–Palade bodies not only increases endothelial permeability by itself but also stimulates the release of angiopoietin-2 from these granules. Vascular endothelial permeability is regulated, in addition to VEGF, by the angiopoietin/Tie2 system. Tie2 is an endothelium-specific transmembrane tyrosine kinase receptor, with angiopoietin-1 and angiopoietin-2 as its competing ligands. While the agonist angiopoietin-1 protects endothelial integrity by strengthening the intercellular junctions, the competitive antagonist angiopoietin-2 increases endothelial permeability [32]. It is possible that following ricin intoxication, during VWF release, there is a secretion of angiopoietin-2 from the endothelium that, upon binding to the Tie2 receptor, is responsible for the reduction in junctional VE-cadherin and intercellular gap formation. An angiopoietin-1 mimetic or inhibition of circulating angiopoietin-2 may, therefore, provide interesting future therapeutic targets to attenuate vascular permeability following systemic ricin poisoning.

The endothelial glycocalyx layer found on the luminal surface of all endothelial cells also plays a crucial role in the preservation of the integrity of the barrier formed by the vessel wall. Accordingly, the critical importance of the glycocalyx has been highlighted in different vascular pathologies, particularly in sepsis or septic shock, where its degradation plays a causative role in vascular barrier dysfunction and organ failure development [33]. Moreover, glycocalyx degradation products, such as soluble syndecans, serve as markers of endothelial dysfunction, sepsis severity and higher mortality [34]. In this study, we show, for the first time, extensive damage of the glycocalyx following systemic ricin intoxication of mice and swine, involving shedding of not only GAG side chains, such as HS and HA, but also of the membrane-bound core proteoglycan, syndecan-1.

Under normal conditions, the endothelium is anticoagulated by the glycocalyx, which is rich in heparonoids, such as antithrombin and thrombomodulin. In addition, it has been shown that VWF is also anchored to the endothelial glycocalyx on the vascular endothelium [35]. Degradation of the glycocalyx and the natural anticoagulant and profibrinolytic factors from the injured endothelium induces the profound hypocoagulability state observed in patients with shock [36]. Following intramuscular ricin intoxication, we detected, in addition to the VWF detachment mentioned above, other biomarkers of endothelial cell injury, such as soluble thrombomodulin and ICAM-1. Moreover, similar to shock models, following ricin exposure, shedding of the glycocalyx was found to coincide with a coagulopathy manifested as prolongation of PT and APTT. These changes were accompanied by multi-organ hemorrhages that may have stemmed from the hypocoagulopathic state of the animals. In septic shock, it has been proposed that the coagulopathy is a compensatory mechanism counterbalancing the shock-induced pro-thrombic vascular endothelium in the microcirculation, in order to secure sufficient organ perfusion in conditions involving shock. Our group has previously shown that systemic exposure of swine to ricin leads to a state of distributive shock, characterized by severe vasodilation and decreased tissue oxygenation evaluated in the macrocirculation [8]. Here, we used SDF intravital microscopy imaging to evaluate microcirculation dysfunction following glycocalyx degradation in ricin-exposed animals. Analysis of sublingual microcirculation by SDF has been recognized as a tool that improves risk stratification or prognostication [37]. We showed that the microvasculature of both mice and swine suffered from ongoing hypoperfusion and increased flow heterogeneity, which worsened progressively with the passage of time. These observations are similar to those found to correlate with mortality in patients with severe sepsis [38]. In this respect, a growing body of evidence indicates that the pathogenesis of sepsis involves an inability to consume oxygen in the peripheral tissues caused by impaired oxygen utilization and mitochondrial dysfunction [39]. These aberrations may play also a critical role in the pathology of systemic ricin poisoning and thus have to be elucidated in the future.

## 4. Materials and Methods

### 4.1. Animals

Experiments were performed in accordance with Israeli law and approved by the Institutional Animal Care and Use Committee (IACUCs) of the Israel Institute for Biological Research. Treatment of animals was in accordance with regulations outlined in the USDA Animal Welfare Act and the conditions specified in the National Institute of Health Guide for Care and Use of Laboratory Animals.

Female CD-1 mice (27–32 g) were purchased from Charles River Laboratories Ltd., Margate, UK. Mice were housed in filter-top cages in an environmentally controlled room and maintained at 21 ± 2 °C and 55 ± 10% humidity. Lighting was set to mimic a 12/12 h dawn to dusk cycle. Female pigs (Topigs 20, 15–24 kg, aged 7–9 weeks) were obtained from an approved commercial source (Van Beek, Netherlands) and fed a standard pig diet. Mice and swine were housed in a purpose-built animal holding facility for 4–8 days prior to the beginning of the experiment. Animals were allowed access to water ad libitum and 4% body weight of food per day. In experiments with swine, 15 hours before the experimental procedure, food was withdrawn, while water remained freely available.

### 4.2. Ricin Preparation and Intoxication

Crude ricin was prepared from seeds of endemic *Ricinus communis*. Seeds were homogenized in a blender (Waring, Torrington, CT) in 5% acetic acid (Merck, Darmastadt, Germany)/PBS (Biological Industries, Beth-Haemek, Israel). The homogenate was centrifuged and the clarified supernatant containing the toxin was subjected to ammonium sulfate (Merck, Darmstadt, Germany) precipitation (60% saturation). The precipitate was dissolved in PBS (Biological Industries) and dialyzed extensively against the same buffer. The toxin preparation appeared on a Coomassie blue (Bio-Rad, Rishon Le Zion, Israel)-stained nonreducing 10% polyacrylamide gel (Thermo Fisher Scientific, Carlsbad, CA, USA) as two major bands with a molecular mass of ~65 kDa (ricin toxin, ~80%) and 120 kDa (*Ricinus communis* agglutinin, ~20%). Protein concentration was determined as 2.86 mg/mL by 280 nm absorption (Nanodrop 2000; Thermo Fisher Scientific).

Prior to intoxication, mice were anesthetized by an intraperitoneal injection of ketamine (1.9 mg/mouse, Vetoquinol, Lure, France) and xylazine (0.19 mg/mouse, Eurovet Animal Health, AD Bladel, The Netherlands). Crude ricin at a dose of 2× median lethal dose (2LD_50_, 18 µg/kg body weight, 1 µL) was applied intramuscularly into the right thigh. Pigs were anesthetized with an intramuscular injection of ketamine and xylazine (20 and 2 mg/kg, respectively) following intramuscular injection of a lethal dose of crude ricin (7.5 µg/kg, 10 µL/kg) into the thigh muscle.

### 4.3. Permeability Analysis

Organ permeability was determined by the Evans Blue dye (EBD) extravasation method as follows: EBD (7.5 mg/mL, Sigma-Aldrich, Rehovot, Israel) was injected intravenously into mice at a dose of 50 mg/kg and allowed to circulate for 1 h. Mice were then anesthetized, and the hearts were perfused through the left ventricle with 5 mL PBS. The heart, spleen and kidneys were removed, and EBD was extracted by incubation of the tissues in 0.5 mL of formamide (Sigma-Aldrich, Rehovot, Israel) at 60 °C for 24 h. The optical density of EBD in the supernatant was measured at 620 nm in a spectrophotometer (Molecular Devices, Sunnyvale, CA, USA) and the total amount of dye was calculated by means of a standard calibration curve.

### 4.4. Blood Analysis for Glycocalyx Shedding and Coagulation

Blood samples collected in microtainers without a coagulant (Beckton Dickenson, Franklin Lakes, NJ, USA) were kept at room temperature (RT) for 30 min to allow clotting, and the serum was separated by centrifugation at 2000× *g* for 1 (mice) or 10 (swine) min. Heparan sulfate (HS), hyaluronic acid (HA), syndecan-1, VEGF, thrombomodulin and ICAM-1 levels were measured using LSBio (Seattle, WA, USA) Mouse Heparan Sulfate ELISA kits (LS-F39210), Porcine Heparan Sulfate Proteoglycan ELISA kits (LS-F39517), R&D Systems (Abingdon, UK), Porcine/Mouse Quantikine Hyaluronan Immunoassay kits (DHYAL0), Diaclone (Besancon Cedex, France) Murine sCD138 (Syndecan-1) ELISA kits (860.090.096), FineTest (Wuhan, China) Porcine SDC-1 (CD138) ELISA kits (EP0231), R&D Systems (Abingdon, UK) DuoSet Mouse VEGF (DY493), Mouse Thrombomodulin SimpleStep (ab209880) and ICAM-1 (CD54) Mouse ELISA kits (ab100688) by Abcam (Cambridge, UK) according to the manufacturers’ instructions. For determination of the clotting parameters, blood was collected using sodium citrate (3.2%, Merck-Millipore, Burlington, MA, USA). After 30 min of incubation at RT, plasma was separated by centrifugation at 2000 g for 10 min. Prothrombin time (PT) and activated partial thromboplastin time (APTT) were determined using TEClot PT and APTT kits on a Coatron M2 instrument (TECO, Cranbury, NJ, USA) according to the manufacturer’s instructions.

### 4.5. Immunohistochemistry and Immunofluorescence

Inguinal lymph nodes (ILNs), livers, kidneys, spleens and hearts were collected and fixed in 4% buffered formaldehyde in PBS pH 7.2–7.4 (Bio Lab, Jerusalem, Israel) for 2 wk. Sections of 5 µm were prepared after paraffin embedding using an RM 2255 microtome (Leica, Wetzlar, Germany) and stained with H&E. Images were captured with a light microscope (Nikon Eclipse E200, Nikon, Melville, NY, USA) coupled with a Digital Sight-Fi1 camera (Nikon, Brighton, MI, USA) using NIS-Elements software (Nikon, Melville, NY, USA).

For VWF, HA, syndecan-1, HS and VE-cadherin staining, sections were deparaffinized in xylene and rehydrated through ethanol gradient solutions to water. Heat-induced antigen retrieval was performed in Target Retrieval Solution (S1700, DAKO, Carpinteria, CA, USA) for 30 min at 95 °C. After blocking in 5% BSA in PBS, slides were incubated overnight at 4 °C with rabbit polyclonal anti-VWF (ab6994), sheep polyclonal anti-HA (ab53842), rabbit monoclonal anti-syndecan-1 (ab128936), rat monoclonal anti-HS (ab2501) and rabbit polyclonal anti-VE-cadherin (ab33168) (Abcam, Cambridge, MA, USA). Alexa Fluor 594-coupled donkey anti-rabbit (ab150155), donkey anti-sheep (ab150180) and goat anti-rat (ab150160) (Abcam, Cambridge, MA, USA) antibodies were used for detection. For ricin binding, sections were incubated with ricin (5 µg/mL) for 30 min. The toxin was then washed, and the slides were incubated overnight at 4 °C with equine-derived anti-ricin antibody (PPII, 0.51 mg/mL, produced in-house). Texas Red-coupled rabbit anti-horse (ab6919, Abcam, Cambridge, MA, USA) antibodies were used for detection. For nuclear staining, slides were mounted with Prolong^®^ Gold antifade reagent containing DAPI (Molecular Probes^®^, Thermo Fisher Scientific, Carlsbad, CA, USA). Analysis was performed using an LSM 710 confocal scanning microscope (Zeiss, Jena, Germany) equipped with the following lasers: argon multi-line (458/488/514 nm), diode (405 nm), DPSS (561 nm) and helium-neon (633 nm).

### 4.6. Microcirculatory Assessment

For microcirculatory monitoring, we used a current videomicroscopic approach: side-stream dark field (SDF) imaging (MicroScan, Microvision Medical, Amsterdam, NLD). During SDF spectroscopy, a handheld video microscope emits stroboscopic green light (540 nm), which is absorbed and immediately emitted back by the hemoglobin of red blood cells. The image of red blood cells moving within the microvessels is transmitted back through the microscope to the camera. Thus, noninvasive real-time images of microcirculation were obtained. For microcirculation measurements, animals were anesthetized by inhalation of ~2% isoflurane in 100% oxygen and then placed on an animal warming pad. At least five video sequences of 7 s duration from different parts of the cecum (mice), or sublingual mucosa (swine) were acquired per time point. The acquired data were analyzed using Micro Vision Medical Automated Vascular Analysis (AVA) (version 4.3C Microvision Medical, Amsterdam, NLD) software. The following parameters were acquired for small vessels (diameter ≤20 µm): total vessel density (TVD), proportion of perfused vessels (PPV), microvascular flow index (MFI) and heterogeneity index (HI). For MFI quantification, the image was divided into four quadrants and the circulation in each quadrant was expressed on an ordinal scale: 3, continuous flow; 2, sluggish flow; 1, intermittent flow; 0, no flow. MFI was the average score of all quadrants. HI was calculated per time point in every animal as follows: maximum MFI minus minimum MFI divided by the mean MFI.

### 4.7. Statistical Analysis

All statistical analyses were conducted with GraphPad Prism software (version 5.01, GraphPad Software Inc., La Jolla, CA, USA, 2007). Data are presented as means ± SEM. For multiple comparisons, one-way analysis of variance (ANOVA) tests followed by Dunnett’s multiple comparisons test were applied. Differences were considered significant at *p* < 0.05.

## 5. Conclusions

**Figure 7:**
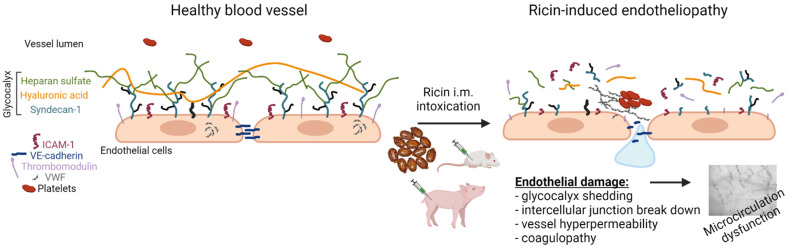
Schematic representation of ricin-induced vascular damage following i.m. exposure of mice and swine. Following ricin intoxication, the vascular endothelium underwent pathophysiological changes, such as degradation of various components of the glycocalyx (syndecan-1, HS and HA), the release of thrombomodulin and ICAM-1, exposure of VWF to the bloodstream and breakdown of VE-cadherin between adjacent endothelial cells. As a consequence of these events, widespread hemorrhages, coagulopathy, vascular hyperpermeability and eventually microvasculature malfunction were detected. Created with BioRender.com.

Collectively, the findings show that systemic ricin intoxication leads to widespread damage of the vascular endothelium, multi-organ hyperpermeability and coagulopathy with microcirculatory dysfunction (Figure 7), and justify the categorization of parenterally induced ricin pathology as a form of shock-induced endotheliopathy [36], a proposed model of disease that unifies the pathological changes observed in acutely critical illness challenged by shock. If so, exploration of therapeutic strategies designed to reduce endotheliopathy may assist to reduce ricin-induced morbidity and enhance the therapeutic performance of anti-ricin antibody therapies [40].

## Data Availability

Not applicable.

## Supplementary Materials

The following are available online at https://www.mdpi.com/article/10.3390/ijms222212345/s1.
