# Supplementary material for: Intramuscular Exposure to a Lethal Dose of Ricin Toxin Leads to Endothelial Glycocalyx Shedding and Microvascular Flow Abnormality in Mice and Swine
Source: Int J Mol Sci. 2021 Nov 16;22(22):12345. doi: 10.3390/ijms222212345 (PMC8618821; doi:10.3390/ijms222212345)
Supplement: Supplementary file 1 [file ijms-22-12345-s001.zip › supplementary figure.pdf]

Suppl. Figure S1

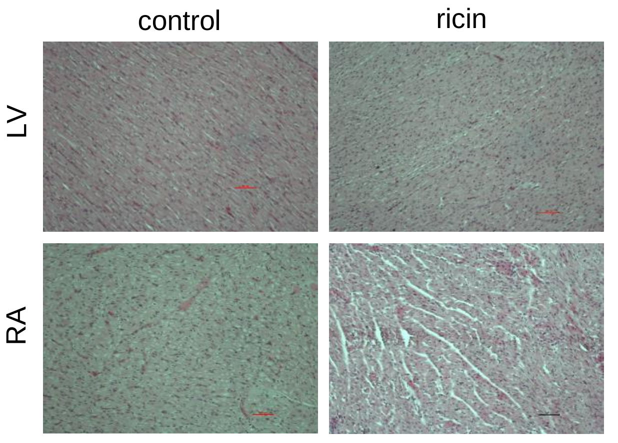

Supplementary Figure S1. Histological analysis of the heart. Representative sections of the heart (left ventricle (LV) and right atrium (RA)) from control and ricin intoxicated swine at 24 h post-exposure (7.5 µg/kg). The sections were stained with H&E. Scale bar: (representative sections of *n*=3 swine/group are shown).

Suppl. Figure S2

Microcirculation movies

control ricin 24h ricin 30h

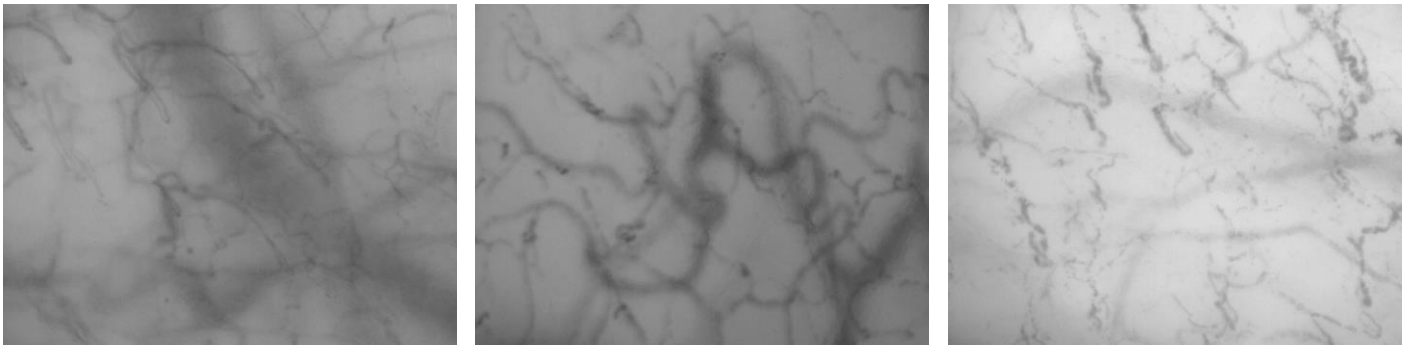

Supplementary Video S1. Sublingual microcirculation in control swine.
Supplementary Video S2. Sublingual microcirculation in swine 24 h after exposure to ricin.
Supplementary Video S3. Sublingual microcirculation in swine 30 h after exposure to ricin.
